# Echocardiography in patients with hypertrophic cardiomyopathy: usefulness of old and new techniques in the diagnosis and pathophysiological assessment

**DOI:** 10.1186/1476-7120-8-7

**Published:** 2010-03-17

**Authors:** Maria-Angela Losi, Stefano Nistri, Maurizio Galderisi, Sandro Betocchi, Franco Cecchi, Iacopo Olivotto, Eustachio Agricola, Piercarlo Ballo, Simona Buralli, Antonello D'Andrea, Arcangelo D'Errico, Donato Mele, Susanna Sciomer, Sergio Mondillo

**Affiliations:** 1 Department of Clinical Medicine, Cardiovascular and Immunological Sciences, University Federico II, Naples, Italy; 2 CMSR Veneto Medica -Altavilla Vicentina, Italy; 3 Department of Clinical and Experimental Medicine, University Federico II, Naples, Italy; 4 Referral Center for Myocardial Diseases, Careggi University Hospital, Florence; 5 Noninvasive Cardiology Unit, Ospedale San Raffaele, IRCCS, Milano, Italy; 6 Cardiology Operative Unit, S. Maria Annunziata Hospital, Firenze, Italy; 7 Department of Clinical Medicine, University of Pisa, Pisa, Italy; 8 Chair of Cardiology, Second University of Naples, Naples, Italy; 9 Azienda Ospedaliera Universitaria, Ferrara, Italy; 10 Department of Cardiovascular, Respiratory and Morphological Sciences, University of Rome, University La Sapienza, Rome, Italy; 11 Department of Cardiovascular Diseases, University of Siena, Italy

## Abstract

Hypertrophic cardiomyopathy (HCM) is one of the most common inherited cardiomyopathy. The identification of patients with HCM is sometimes still a challenge. Moreover, the pathophysiology of the disease is complex because of left ventricular hyper-contractile state, diastolic dysfunction, ischemia and obstruction which can be coexistent in the same patient. In this review, we discuss the current and emerging echocardiographic methodology that can help physicians in the correct diagnostic and pathophysiological assessment of patients with HCM.

## Introduction

Hypertrophic cardiomyopathy (HCM) is clinically defined in presence of left ventricular (LV) hypertrophy in the absence of hypertension and valve disease. LV hypertrophy without cardiovascular causes occurs in approximately 1:500 of the general population [1-3]. This incidence includes all kinds of hypertrophy not necessarily HCM, which is a familial disease with an autosomal dominant pattern of inheritance caused by mutations in genes encoding for sarcomeric proteins resulting usually in an asymmetrical pattern of LV hypertrophy. Echocardiography plays a pivotal role in detecting the disease and understanding its pathophysiology. In this review, we discuss the current and emerging echocardiographic methodology that can help physician in the correct diagnosis and pathophysiological assessment of patients with HCM.

## Echocardiography and Diagnosis

### Conventional Echocardiography

HCM may be initially suspected because of an heart murmur, positive family history, new symptoms or abnormal ECG pattern showing LV hypertrophy and abnormal Q waves. Thereafter an echocardiogram is usually performed.

#### M-Mode Echocardiography

**Figure 1:**
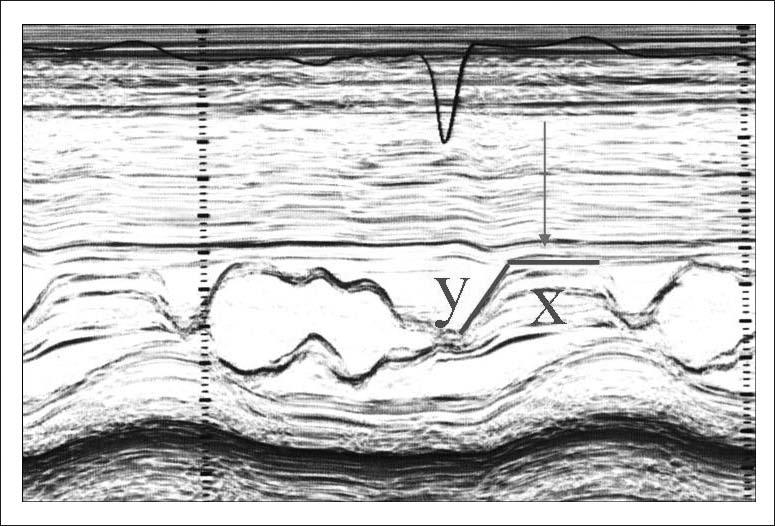
**Systolic anterior motion (SAM) of the mitral valve visualized by M-Mode echocardiography**. X represent the duration of SAM, whereas Y represent the time elapsed between the beginning of SAM and the SAM-septal contact (see text for more details).

The first echocardiographic diagnostic criteria in HCM were established by using M-mode imaging which included asymmetrical septal hypertrophy, systolic anterior motion of the mitral valve (SAM), a small LV cavity, septal immobility, and premature closure of the aortic valve [1-3]. LV thickness, evaluated at septum and free wall level, is considered abnormal when ≥ 15 mm, and defined asymmetrical in presence of a septal to free wall thickness ratio between 1.3 and 1.5. SAM is characterized by an abrupt anterior movement of the mitral valve reaching its peak before maximum movement of the posterior wall (Figure 1); this characteristic allows to differentiate true SAM from SAM produced by an exaggerated anterior motion of the mitral valve which reaches its peak after the fully contraction of the posterior wall, i.e. "pseudo SAM" [4]. There is a positive correlation between the severity of SAM and the severity of obstruction evaluated invasively [5]. Usually, a contact between SAM and the septum indicates an obstruction ≥30 mmHg. Moreover, the measurement of the time interval from the beginning of SAM to the SAM-septal contact (y) and the duration of SAM-septal contact (x) provides a reliable non-invasive method for estimation of the pressure gradient, where the gradient is (x/y)*25+25 mmHg (Figure 1) [5].

The movement of mitral valve is easily visualized by M-Mode and its anterior motion during systole, together with asymmetrical septal hypertrophy, was initially thought to be pathognomonic of HCM. However, these findings may be present in other forms of secondary and primary hypertrophy, like chronic systemic and reno-vascular hypertension [6], Fabry's disease [7,8], glicogenosis [9], Friedreich ataxia [10], etc.

Left atrial (LA) diameter is usually increased in patients with HCM because of obstruction and/or diastolic dysfunction. LA diameter gives informations about the risk of atrial fibrillation, of heart failure development and of cardiac mortality [11] which is particularly high in patients with a LA diameter > 48 mm. Finally, LA fractional shortening, evaluated as ([maximal diameter-minimum diameter]/maximal diameter*100), is an estimate of end-diastolic pressure in HCM [12]; this parameter is directly related to exercise tolerance [13], and its reduction (i.e.<16%) represents an independent risk factor for atrial fibrillation development [14].

#### Two-dimensional echocardiography

This technique by visualizing the whole heart has come the recognition that LV hypertrophy is most often asymmetrical and it can be confined in specific LV segments such as the apex.

### LV hypertrophy and function

**Figure 2:**
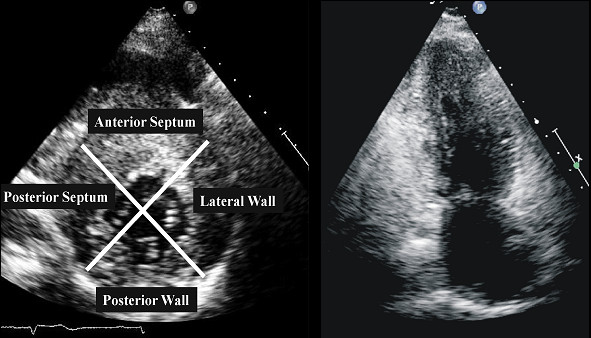
**Left ventricular walls dived in four regions in a patients with HCM**. ***Right panel***. Apical view in a patient with apical hypertrophy.

**Figure 3:**
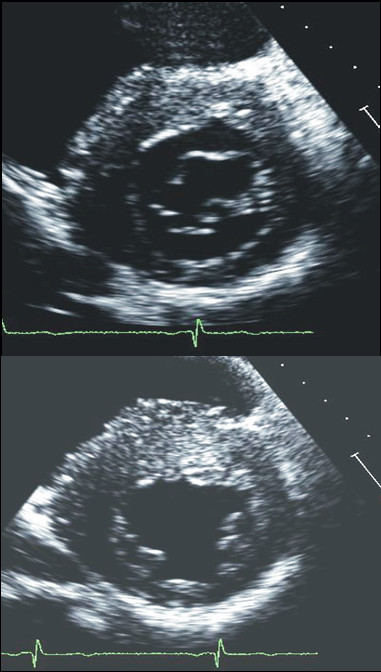
**Examples of patients with hypertrophic cardiomyopathy with typical asymmetrical left ventricular hypertrophy**.

**Figure 4:**
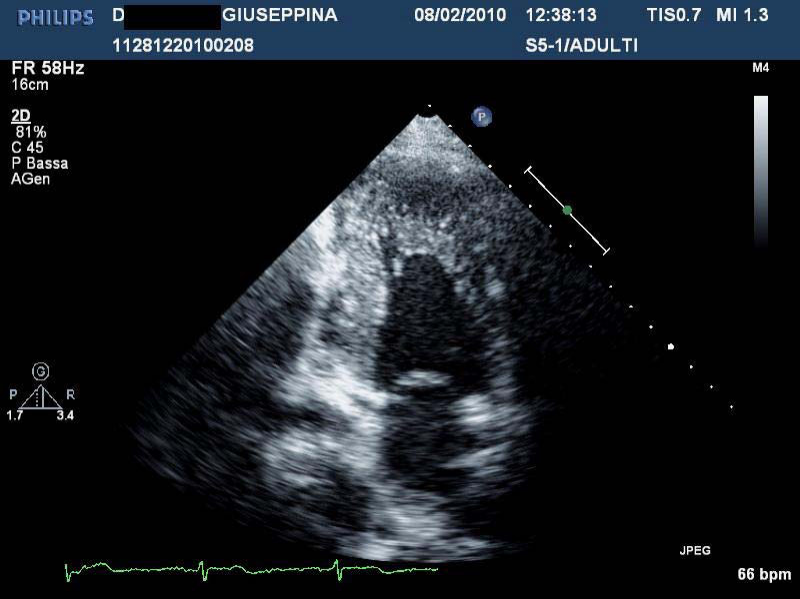
**Patient with left ventricular non compaction**. This patient was sottoposed to echocardiography in the contest of family screening for apical hypertrophic cardiomyopathy.

Currently, using short-axis view the left ventricle is divided in 4 LV wall segments: anterior and posterior septum and posterior and lateral wall (Figure 2, left panel) [4]. Segments are visualized at mitral and papillary level, whereas the possible extension to the apex is visualized by 4 chamber view (Figure 2, right panel). Classical LV hypertrophy cut-off suggestive of HCM in the general adult population is 15 mm [15]. Usually the pattern of LV hypertrophy is asymmetrical, with the anterior septum involved in the majority of cases being also the site of the maximal LV hypertrophy in most patients (Figure 3). In almost 40% of patients, LV hypertrophy involves two segments, whereas the concentric pattern or hypertrophy confined to the apex are particularly uncommon in Western countries (1% each) [16]. Recently, it has been demonstrated [17] that mutations in the alpha-cardiac actin gene can express apical HCM or LV non compaction or septal defects. Nevertheless, LV non compaction has to be differentiated from the apical form of HCM (Figure 4).

**Figure 5:**
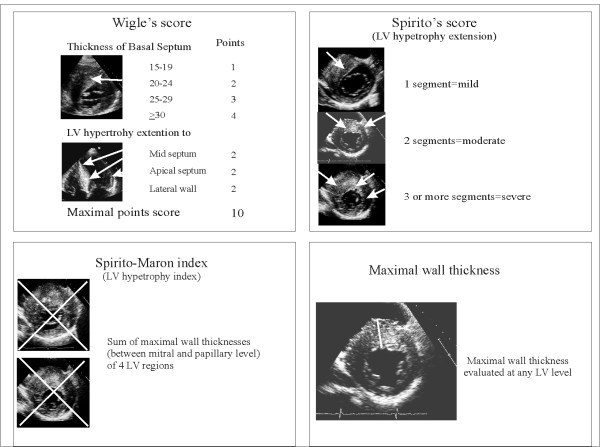
**Echocardiographic methods to identify the degree and the extension of left ventricular hypertrophy in patients with hypertrophic cardiomyopathy**. LV = left ventricular

The heterogeneous distribution of hypertrophy in HCM results in a distortion of internal LV shape allowing algorithms, generally used to measure LV mass, not applicable in this disease. As a consequence, several echocardiographic indexes have been developed to measure the distribution and the extent of LV hypertrophy. Wigle et al [18] proposed a points score system which takes into account the degree of septal thickness, starting from a value of 15 mm, and the extension of hypertrophy up to the point of the apex. In order to calculate this score, the apical 4- chamber view is used to determine the extent of septal involvement, and the parasternal short-axis view at level of the mitral valve leaflet tips to determine the anterolateral wall involvement (Figure 5). Also, Spirito et al [15] have developed a system for assessing the magnitude of hypertrophy using the parasternal long and short-axis views and apical views (Figure 5). The overall extent of hypertrophy is defined as mild if only one LV segment is involved, moderate if two segments are involved and severe if three or more segments are involved [15] (Figure 5). Moreover, an index of hypertrophy, the Spirito-Maron index, is obtained by adding the maximal wall thickness of each LV segments. The most clinical important method is the measurement of the maximal wall thickness (MWT) at any LV level [19] (Figure 5). Extreme wall thickness, i.e. ≥ 30 mm, which can be detected at any site of LV wall, is observed less commonly in older than in younger patients, probably because of sudden death (SD) at a young age and/or of structural remodeling with wall thinning increasing with age. Spirito et al [20] showed that a maximum thickness of 30 mm or more, present in approximately 10% of HCM patients, resulted in a substantial long-term risk. However, Elliot et al [21] suggested that extreme hypertrophy is a predictor of SD only when associated with other risk factors such as unexplained syncope, family history of premature SCDs, non-sustained ventricular tachycardia at at Holter-ECG, or an abnormal blood pressure response during exercise. Furthermore, Olivotto et al [22] in a community-based population with HCM reported, during 12-year follow-up, association between maximum LV thickness and SD only in patients diagnosed at a very young age.

The degree of LV hypertrophy varies throughout life. In fact, although the gross phenotypic expression and clinical profile of HCM may occasionally be identified in infants and young children, marked LV hypertrophy is rarely documented during the first years of life [23]. Conversely, rapid changes in LV morphology often occur during adolescence and are frequently delayed completely until the second decade of life, when LV wall thickness may increase rapidly.

Genetic studies among large families demonstrated that morphological LV hypertrophy reaches a plateau at the third decade of life in β-myosin heavy chain and in α-tropomyosin mutations, whereas it increases continuously thought life in cardiac myosin binding protein C mutations [24]. The clinical implication of such an observation is that, in the contest of family screening, repeat echocardiogram to identify LV hypertrophy at yearly intervals is reasonable during adolescence, whereas repeat imaging is considered for adults at longer time intervals of 5 years. All myocardial segments, and not only the interventricular septum, should be carefully examined for screening purposes. In a recent study where genotyping was the gold standard, the Spirito-Maron hypertrophy score was highly specific with a better sensitivity than MWT [25].

**Figure 6:**
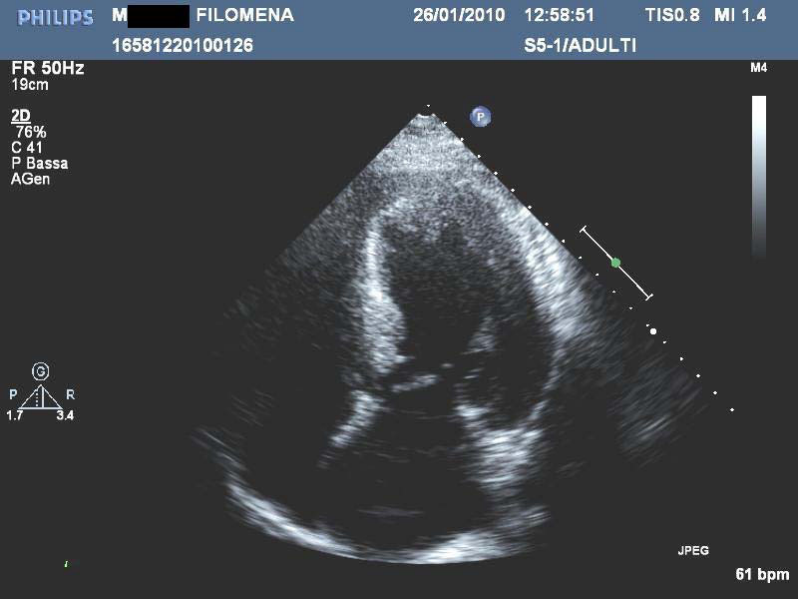
**Patient with end stage hypertrophic cardiomyopathy: note the absence of thinning: the left ventricular ejection fraction is 40%**.

Although LV remodeling in children is characterized by progression of hypertrophy, the changes in cardiac morphology observed in some adults with HCM occur in the context of development of systolic dysfunction (defined as LV ejection fraction <50%) associated or not to LV wall thinning [26] (Figure 6). This unfavorable evolution in the natural history of HCM usually develops during midlife in about 4% of patients [27]. This phase defined end stage (ES) HCM is a cause of progressive heart failure and is characterized by substantial cardiac remodeling and gradual evolution from the typical hypertrophied, non-dilated, and hyper-dynamic state to one of systolic dysfunction. ES diagnosis is primarily dependent on ejection fraction <50%, and ES commonly does not present as a dilated cardiomyopathy, with only almost 50% of patients showing associated LV cavity enlargement or regression in wall thickness; a small proportion of ES patients even demonstrate persistent marked hypertrophy with non-dilated left ventricle [27]. Clinical course is variable but generally unfavorable, and vigilant follow-up are required for timely identification of transition to ES, in order to establish appropriate pharmacological treatment for systolic pump failure, implantable defibrillator for sudden death prevention, heart transplantation.

Cross-sectional echocardiographic analyses of large HCM patient populations encompassing a broad age spectrum [28,29] have documented morphologic differences between youthful and older patients indicating that gradual LV remodeling involving some degree of wall thinning may occur slowly over decades and may be an obligatory pathway in the natural history of HCM [28]

At opposite site, there is evidence of high degree of hypertrophy suggestive of HCM in elderly patients with and without history of arterial hypertension [30,31]. In these patients genetic and family screening are recommended, although technique such as Tissue Doppler Imaging (TDI) and/or strain rate imaging may help in the differential diagnosis as reported below [32].

Finally, among the spectrum of sarcomeric contractile protein disease, idiopathic restrictive cardiomyopathy is part of the clinical expression of cardiac troponin I mutations [33].

### LV hypertrophy in other forms of genetic diseases

Echocardiography can visualize thickened LV walls with high sensibility and specificity, however it cannot distinguish conditions based on myocyte hypertrophy from those in which LV mass and wall thickness are increased by interstitial infiltration or intracellular accumulation of metabolic substrates. Cardiac magnetic resonance (MRI) may help into diagnostic iter (see below), moreover the final diagnosis is given only in some specific conditions by myocardial biopsy, and in particular is not indicated for the final diagnosis of HCM [34,35]. On the other hands, it is not reasonable to investigate for each entity capable of induce increased wall thickness because of anamnestic, clinical and instrumental data that serve to orient the diagnosis before the echocardiographic study.

**Figure 7:**
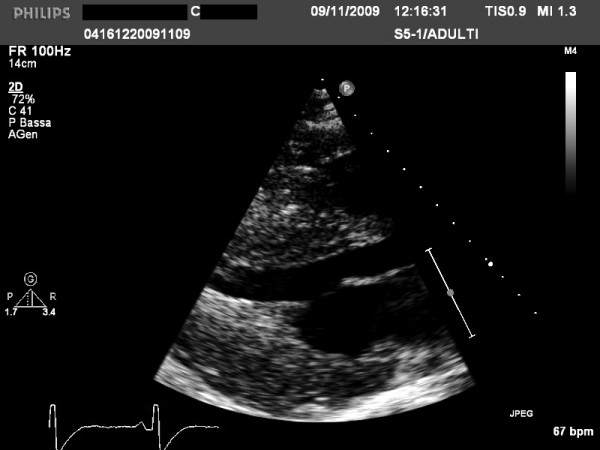
**Patient with extreme left ventricular symmetrical hypertrophy**. At electrocardiography patient showed a short PR. The patient was referred for aminotransferase and creatinine phosphokinase dosage and for genetic testing.

In the contest of genetic disease, thickening of LV walls can results also by mutations in non sarcomeric proteins involving or the gene encoding the γ-2-regulatory subunit of the AMP-activated protein kinase (PRKAG2), or the gene encoding lysosome-associated membrane protein 2 (LAMP-2), resulting in Danon-type storage disease with clinical manifestations limited largely to the heart (usually with massive degrees of LV hypertrophy and ventricular pre-excitation) [9] (Figure 7). Overall these condition are X-linked, and, thus, important clinical clues are male gender and young age.

**Figure 8:**
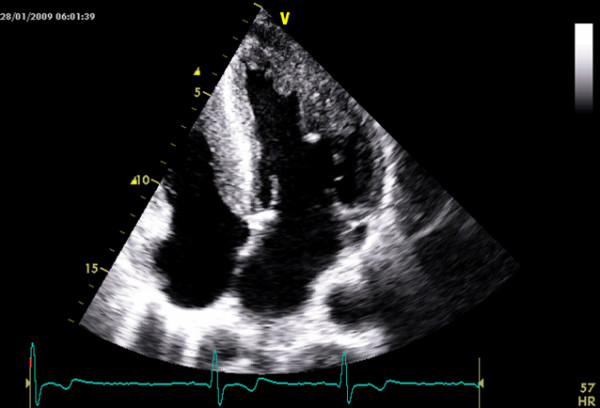
**Young male with definitive diagnosis of Anderson- Fabry disease**. For courtesy of Dr L. Spinelli.

Anderson-Fabry disease is a relatively frequent cause of idiopathic LV hypertrophy. It is a X-linked lysosomal storage disorder caused by α-galactosidase mutations and it is characterized clinically by widespread variety of signs and symptoms. Cardiac findings include LV hypertrophy, showing a symmetrical pattern in the majority of cases, mild diastolic dysfunction and preserved LV ejection fraction as well as no LV outflow tract obstruction (LVOTG) (Figure 8). The use of a binary appearance at echocardiography of LV endocardial border has been questioned in that is not a sensitive marker and it can not be routinely used to differentiate Anderson-Fabry disease from HCM [7,8]. Symptomatic cardiac involvement usually occurs in most affected males, whereas female carriers present with minimal or no symptoms. Retrospective studies found a prevalence of Anderson-Fabry disease in 4-6% of patients previously classified as HCM, suggesting that the disease should be suspected in male patients with concentric LV hypertrophy and no family history of HCM or with inheritance consistent with X-linked disease [36].

**Figure 9:**
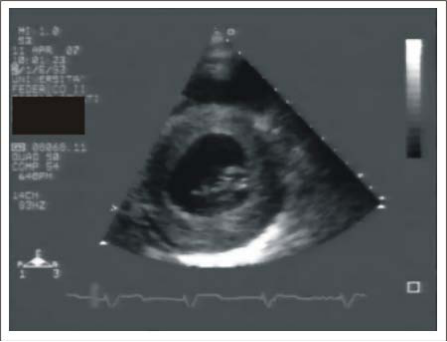
**Patient with definite diagnosis of mithocondropathy done by neurologists**. Echocardiography shows mild symmetric hypertrophy with mild reduction of left ventricular ejection fraction. During follow-up patient developed severe grade of AV block needing pace-maker and severe reduction of ejection fraction needing resynchronization.

Mitochondrial disorders result from abnormalities in mitochondrial DNA and function; mitochondrial DNA is inherited maternally, and most of these disorders are transmitted from mother to children of both sexes. In some mitochondrial disorders, LV concentric hypertrophy is present as well dilated cardiomyopathy, which probably represents a progression from the hypertrophic form. In most patients, conduction abnormalities are present [37] (Figure 9).

**Figure 10:**
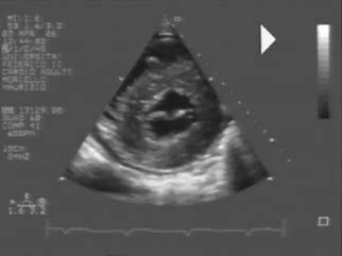
**Patient with definitive diagnosis of amyloidosis**. Note the pericardial effusion.

Involvement of the heart is a common finding and is the most frequent cause of death in amyloidosis; cardiac amyloidosis occurs more commonly in men than in women, and it is rare before the age of 40 years. The onset of clinical cardiac disease usually occurs late in life. Echocardiography is characterized in the majority of cases by symmetric LV hypertrophy, dilated atria and pericardial effusion (Figure 10). In some case the degree and the distribution of hypertrophy may resemble HCM, however LV hypertrophy together with the evidence of low voltage at electrocardiogram help in the differential diagnosis with pericardial effusion and with HCM [38].

### Athletic heart and HCM

One of the most discussed issue is how to role out HCM in athletes. Diagnosis of HCM in athletes is important, given the high propensity to sudden cardiac death in HCM patients engaging in competitive sports as well as other physically intense activity [39]. Although in Italy the ECG, which is routinely performed in competitive athletes, has dramatically reduced the incidence of sudden death due to the identification of diseases such as HCM [40], sometimes the diagnosis can be still particularly challenging in athletes with an advanced degree of physiologic LV hypertrophy. Cardiac hypertrophic response to training is different between sports [41], between individuals of the same race undergoing the same training [42], and is different between races [43]; thus, there is a very huge variability in LV hypertrophy in athletes, which sometimes is suggestive of HCM. Helpful clues include the presence of wall thickness >12 mm in the presence of a non-dilated LV in HCM, because HCM patients usually have normal or reduced LV dimensions and no cavity dilatation (>55 mm is common in athletes), except with disease progression and systolic dysfunction. HCM patients have abnormal myocardial function as detected by TDI, including mitral annulus velocities or strain rate [44]. In equivocal cases, it is reasonable to recommend stopping exercise with repeat imaging later, when one would expect regression of physiologic but not pathologic LV hypertrophy [44,45].

**Figure 11:**
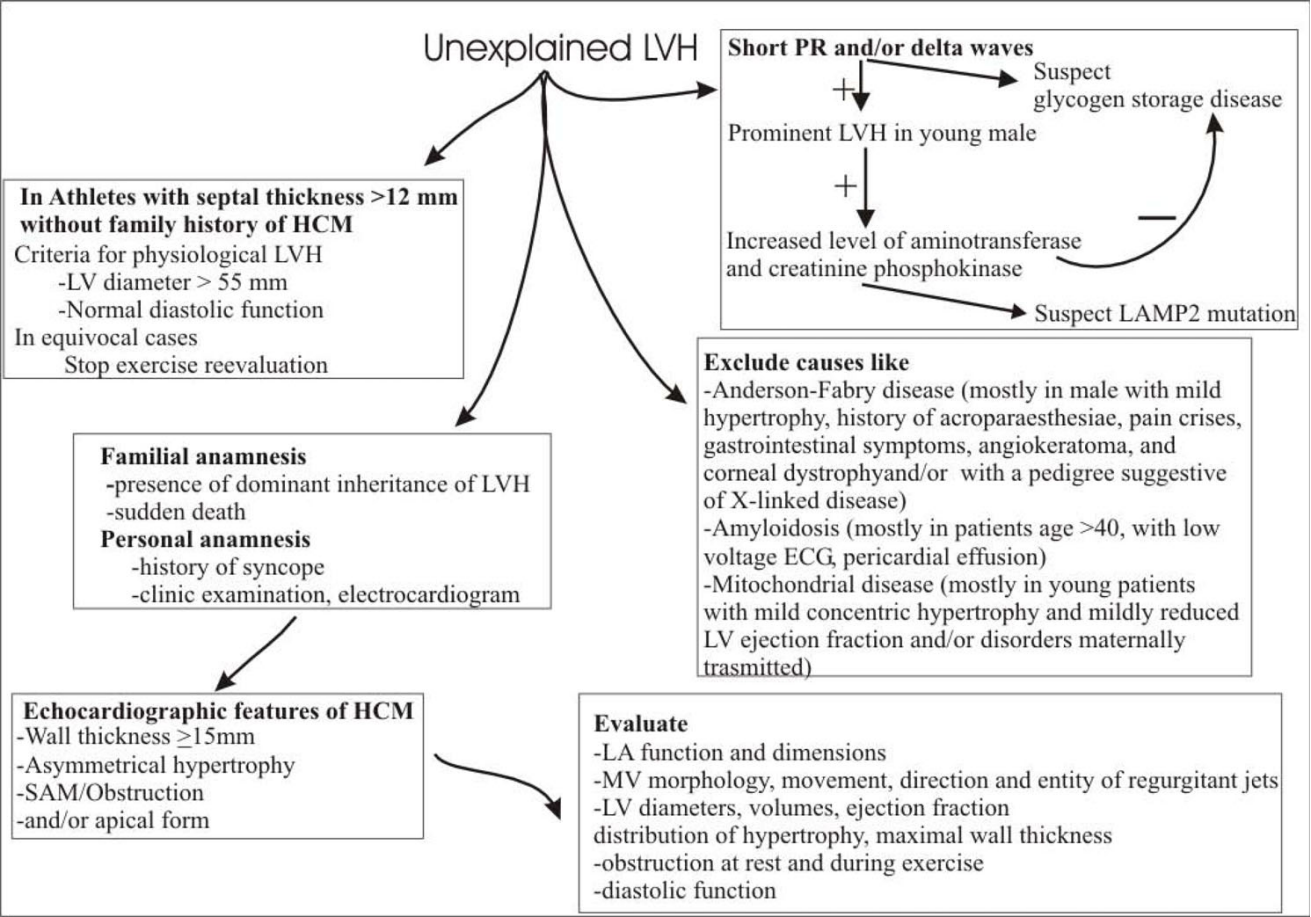
**Scheme for the clinical and echocardiographic approach in patients with unexplained left ventricular hypertrophy**. LV = left ventricular

In Figure 11 we suggest a clinical and echocardiographic method to approach patients with unexplained LV hypertrophy.

### Right ventricular hypertrophy

Right ventricular hypertrophy is diagnosed when two or more right ventricular segments are hypertrophied and when at least two right ventricular wall measurements exceed two standard deviations from the mean recorded in normal subjects. Using these criterions McKenna et al [46] reported right ventricular hypertrophy in 44% of 73 patients with HCM. More recently, Maron at al found [47] in 46 HCM patients studied by cardiac MRI, that right ventricular mass was increased in the majority of them. To date, the clinical and prognostic significance of right ventricular hypertrophy is not known.

### LVOTG

**Figure 12:**
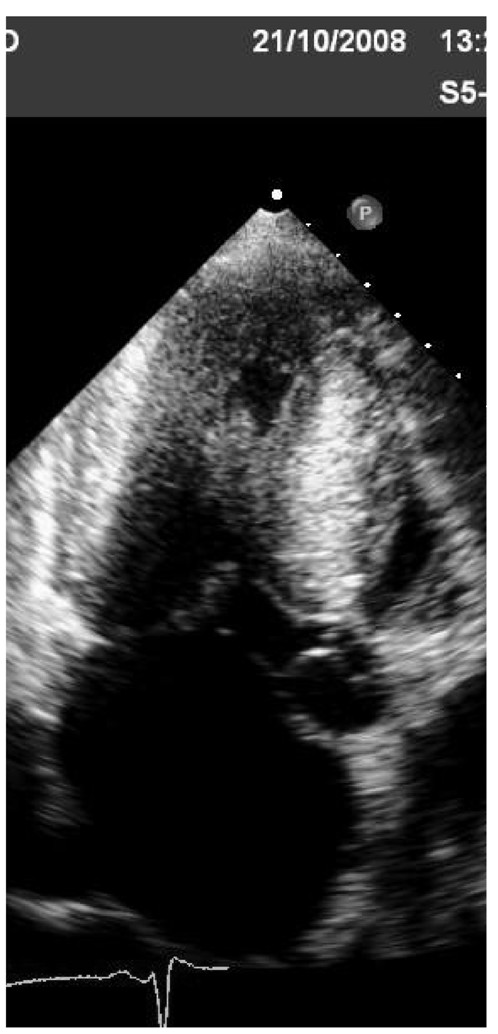
**Mid ventricular obstruction with an hourglass appearance**.

**Figure 13:**
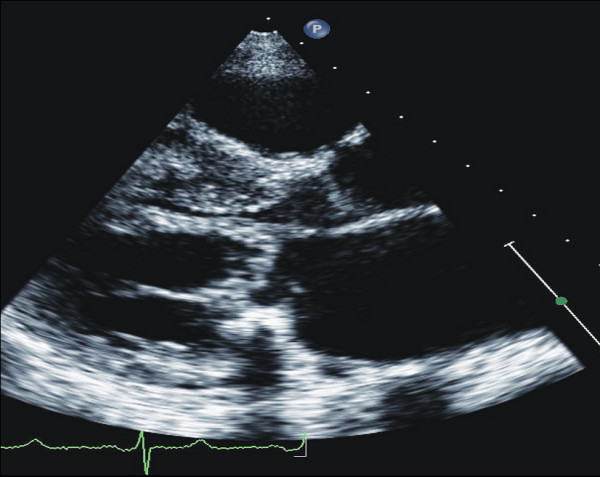
**Example of patients with abnormal direct insertion of papillary muscles into anterior mitral leaflet**.

Although SAM is the most frequent mechanism of LVOTG, obstruction can occur at mid-ventricular level or at multiple levels in the same patient and is variable with time. Continuous Doppler will give informations about the degree of obstruction (see later) whereas by pulse wave Doppler LV mapping will be performed to determine the site of obstruction. Mid-ventricular obstruction has been diagnosed by the typical angiographic feature of hourglass appearance of the left ventricle with mid-ventricular obliteration and apical chamber that is variable in size and contractility. Echocardiography has the same potential to identify this haemodynamic type of HCM (Figure 12). Patients with mid-ventricular obstruction are at high risk to develop segmental, like apical aneurysm, or diffuse LV wall motion abnormalities [48]. Moreover, structural abnormalities of the mitral valve, such as increased mitral valve area and abnormal direct insertion of papillary muscles into anterior mitral leaflet (Figure 13) can be detected by echocardiography orienting treatments' strategies.

### LA volume

LA volume measured by two-dimensional echocardiography has some clinical and prognostic implications. LA remodeling, measured by LA volume, relates directly with exercise tolerance as demonstrated by Sachdev et al [49] in patients without LVOTG either at rest and during provocation, suggesting that this parameter may serve as a surrogate marker of chronic diastolic burden. Moreover, patients with normal LA volume which show dilatation during follow-up, i.e. >3 ml/year, have a worse outcome than patients with normal and stable LA volume during follow-up and similar to that of patients with LA dilation at baseline [50].

#### Doppler echocardiography

Each Doppler technique offers relevant contribution in the analysis of patients with HCM .

### Color Doppler echocardiography

Evaluation of the presence and degree of mitral regurgitation is performed by color Doppler echocardiography. Mitral regurgitation occurs in almost all patients with obstructive HCM as a consequence of SAM which induces abnormal mitral leaflet coaptation and may be an important cause of dyspnea. When additional mitral valve abnormalities other than SAM are not observed, a direct relation between the pressure gradient and the severity of MR is evident [51]. The direction of the mitral regurgitation jet is useful in identifying patients with independent mitral disease. In fact SAM induces a mitral regurgitation jet directed posteriorly, whereas in presence of a intrinsic mitral valve disease due to annular, papillary or leaflet disease, patients with obstruction and mitral regurgitation can show a systolic mitral anterior directed jet [51].

### Continuous Doppler echocardiography. Exercise echocardiography

**Figure 14:**
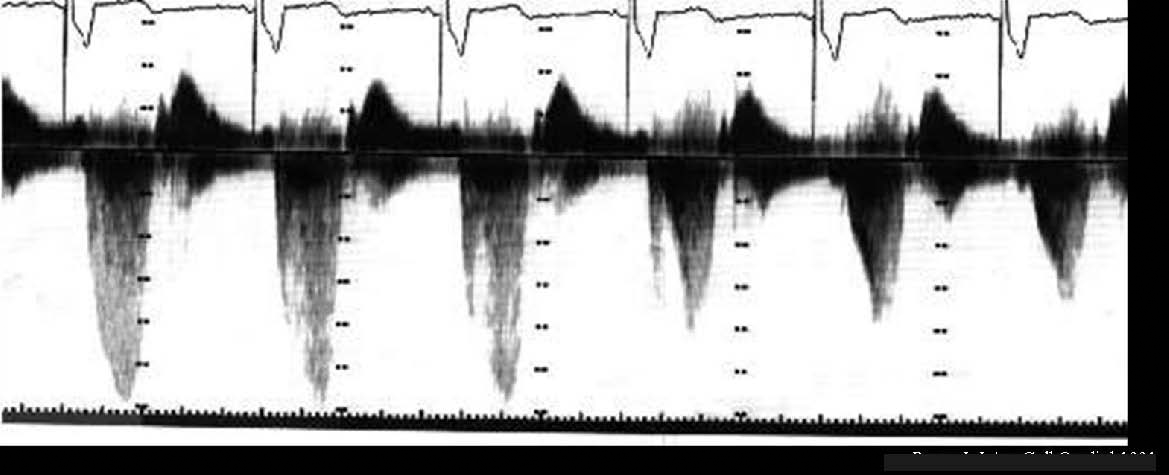
**Differentiation between mitral regurgitation (first cardiac cycles) and left ventricular outflow tract gradient (last cardiac cycles)**. This was obtained by orienting the probe more medially and anteriorly.

Approximately 25% of patients with HCM have a significant resting pressure gradient, i.e. ≥30 mmHg, between the body and LV outflow tract. This is nearly always accompanied by SAM. Continuous wave Doppler is used to determine peak LVOTG with caution exercised to exclude the mitral regurgitation jet [52] (Figure 14). This latter differentiation may be difficult especially in patients with mitral regurgitation jet directed anteriorly. In these cases it is of help the M-mode echocardiographic evaluation of SAM.

It is well recognized that some patients without outflow obstruction at rest have gradients that can be provoked by physiological and pharmacological interventions that diminish LV end-diastolic volume or augment LV contractility. The term labile obstruction has been used to describe the spontaneous appearance and disappearance of obstruction and latent obstruction to describe gradients that only appear with provocation. A number of methods can provoke obstruction in the echocardiography laboratory, including Valsalva maneuver, amyl nitrite, and dobutamine. However, these methods are not standardized and can underestimate the degree of obstruction such as Valsalva maneuver or have low specificity such as dobutamine. Exercise is a physiologic means of provoking latent LVOTG. Over 50% of HCM patients without significant outflow tract obstruction at rest will demonstrate outflow gradients over 30 mmHg with exercise [53,54]. Although supine bike exercise is more conducive to acquiring multiple haemodynamic data sets, this position increases venous return and might decrease the likelihood and extent of LVOTG. Accordingly, upright exercise, which has the greatest resemblance to daily physiologic activities, should be used.

Right ventricular outflow tract obstruction may coexist with LVOTG in a minority of patients with massive septal hypertrophy and occasionally it is isolated [55].

**Figure 15:**
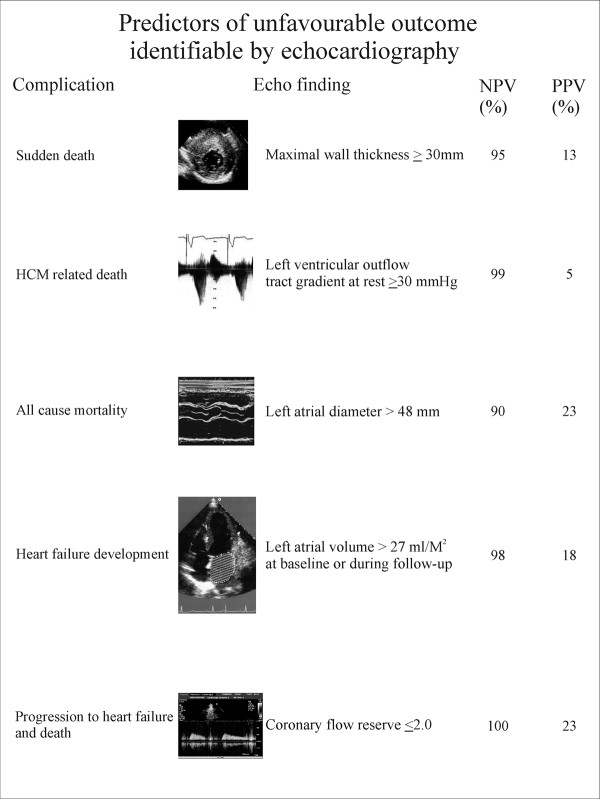
**Echocardiographic findings and their prognostic impact in patients with HCM**.

Figure 15 shows the echo findings which are strong predictors of prognosis in patients with HCM.

### Pulsed Doppler

**Figure 16:**
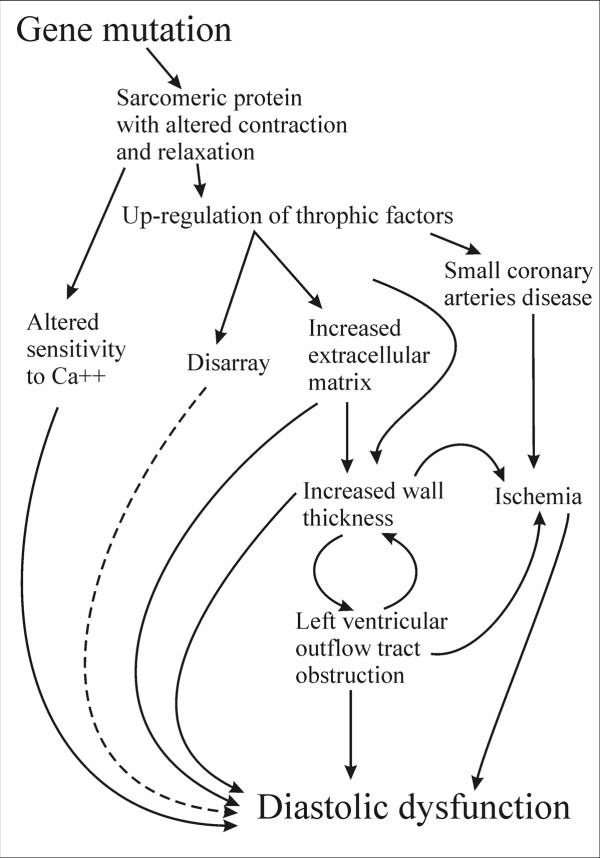
**Mechanisms linked to diastolic dysfunction in patients with hypertrophic cardiomyopathy**. Dashed lines represent links not yet well demonstrated.

Pulsed Doppler at mitral and pulmonary level is used to assess the presence and the degree of diastolic dysfunction. Almost all patients with HCM have some degree of LV diastolic dysfunction. Figure 16 reports a scheme of the molecular, morphological and haemodynamic factors which may contribute to diastolic dysfunction in HCM. These complex mechanisms determine that all phases of diastole are altered. Isovolumic relaxation is slowed and prolonged, the rate of rapid filling is diminished, atrial contribution to filling is increased as well as LV chamber stiffness [56]. The importance of diastolic dysfunction in HCM has led to an extensive search for accurate, noninvasive methods of quantifying its severity. When LV end-diastolic pressure is considered, the echo pulsed Doppler parameter with a good relationship is represented by the difference in duration between the atrial contraction wave at mitral and pulmonary level [57]. Lombardi et al [58] demonstrated that as the difference in duration worsens myocardial collagen synthesis prevails over degradation; moreover, an echocardiography index of myocardial fibrosis, i.e. diastolic back scatter, increases [59], suggesting a strong interplay between diastolic function and myocardial fibrosis.

### New echocardiographic technologies

New technologies have been employed in the patho-physiological assessment, in preclinical diagnosis, in differential diagnosis, and in risk stratification of HCM.

#### Contrast echocardiography

Contrast echocardiography currently is used to enhance endocardial definition, Doppler signals, and to evaluate myocardial perfusion during percutaneous transluminal septal myocardial ablation (PTSMA). PTSMA is a catheter interventional treatment which involves the introduction of absolute alcohol into a septal perforator branch of the left anterior descending coronary artery to produce a myocardial infarction within the proximal ventricular septum. The aim is similar to that of myotomy-myectomy, i.e. reducing the basal septal thickness and excursion enlarging the LV outflow tract and, thereby, lessening the SAM of the mitral valve and mitral regurgitation. The introduction of the echo contrast has proved to reduced the side effects of the technique: it selects the appropriate septal perforator branch determining the precise area of septum targeted for alcohol ablation and evaluates whether selected septal perforator also perfuses other distant and unwanted areas of LV or right ventricular myocardium or papillary muscles [60].

A potential role for contrast enhancement in the diagnosis of apical HCM has been demonstrated [61], although systematic studies have not be yet performed.

#### Tissue Doppler Imaging

Given the complex interplay of factors causing diastolic dysfunction in HCM, it should not be surprising that no single non-invasive measure has been definitively validated. Nagueh et al [57] suggested that the ratio of early transmitral (E) to tissue Doppler early diastolic (e') velocities of the lateral mitral annulus accurately quantified LV pressures, in particular the LV pressure before atrial contraction, an E/e' ≥10 showed the best sensitivity and specificity for identifying LV pre-A pressure > 15 mmHg. However, that ratio shows only a modest correlation when related to mean left atrial (LA) pressure, and, moreover, the predictive accuracy of the E/e' ratio for estimation of mean LA pressure in an individual patient was modest [62]. However, in some study this parameter identifies patients with low exercise capacity [63,64].

TDI has been investigated in the preclinical diagnosis of HCM. Studies from transgenic animal models revealed some abnormal myocardial function at a time preceding the development of LV hypertrophy, firstly due to alterations in Ca^++ ^sensitivity [65] which probably induce low TDI velocities at annular mitral level. Although some reports have provided encouraging results [66,67], additional data from a larger number of subjects are needed to determine TDI velocity values that provide the highest diagnostic accuracy.

#### Strain rate imaging

Patients with HCM may show regional differences in wall motion at rest [45]. Betocchi et al [68] demonstrated that LV regions with less pronounced myopathic process are those with normal stiffness and with supernormal wall motion. In contrast, the stiffer septum shows reduced wall motion compared with adjacent regions. Other mechanisms may explain regional asynergy such as anatomic nonuniformity, altered calcium handling, subendocardial ischemia and altered glucose metabolism.

**Figure 17:**
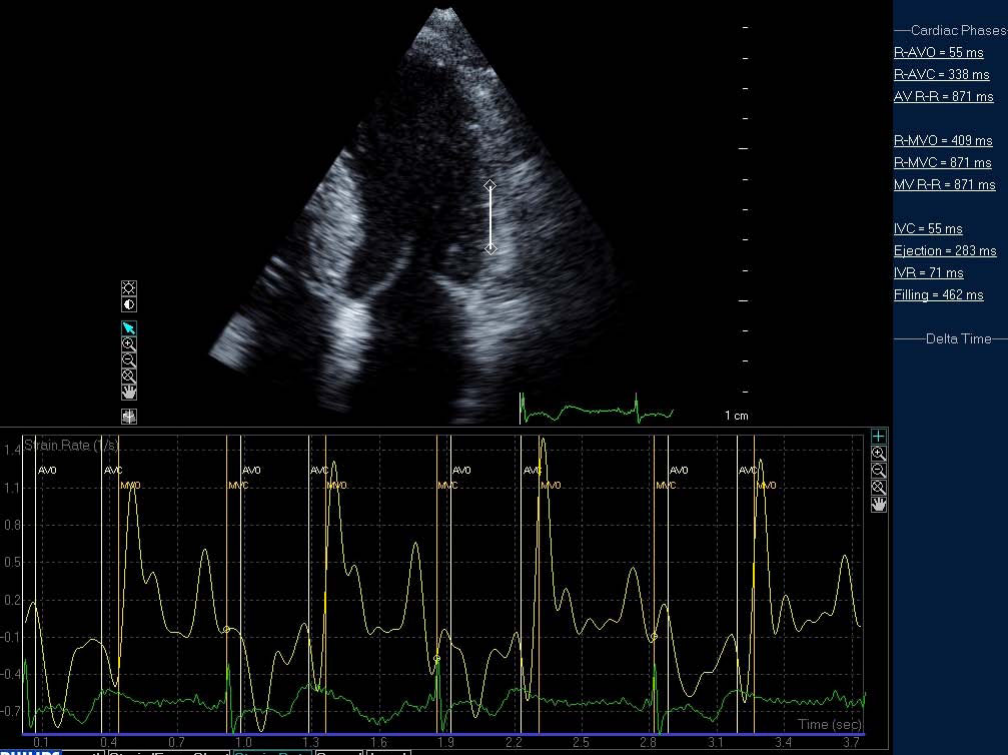
**Example of post-systolic strain in a patient with HCM**.

In the last decade several papers have been published using strain rate technique, either TDI and speckle (2D gray-scale method), to investigate regional systolic function. Ganame et al [69] demonstrated in a pediatric population with HCM that, despite normal global systolic function, longitudinal and radial systolic myocardial deformation were heterogeneously reduced and the alteration was more pronounced in the more severely hypertrophied myocardial segments. There are discrepancies between measures of regional and global myocardial functions for several reasons. Endocardial indexes of LV function such as fractional shortening and ejection fraction are known to overestimate systolic function in the presence of LV hypertrophy. Moreover, patients with HCM have a smaller end-diastolic diameter and increased wall thickness, resulting in a decreased ventricular afterload which, in presence of significant hypertrophy, will result in higher values of fractional shortening and ejection fraction, despite reduced wall thickening. When regional systolic function was studied by strain rate imaging, there was a direct relationship between systolic deformation and exercise capacity in a pediatric population with HCM, suggesting that systolic function, even when ejection fraction is normal or supernormal, has a role into determination of clinical status in these selected group of patients. In another study, a regional double peak systolic sign indicating a second systolic peak during systole, had strong relationship with the late enhancement at nuclear magnetic resonance, suggesting the possibility to diagnose regional myocardial fibrosis by echocardiography [70] (Figure 17).

Strain rate imaging has been shown to measure accurately LV torsion [71]. LV untwisting is linked temporally with early diastolic base-to-apex pressure gradients, enhanced by exercise, which may assist efficient LV filling. This effect appears blunted during exercise in HCM [72], particularly in patients with the obstructive form [73].

A frequent clinical issue is to establish the final diagnosis in patients with high degree of LV hypertrophy and arterial hypertension; recently, more severely reduced systolic compression (by strain Doppler echocardiography) along with asymmetric LV hypertrophy readily identified HCM patients from those with hypertension [32]. Receiver-operator characteristic curve analysis identified the optimal cut-off value of strain, i.e. systolic longitudinal strain by 4 and 2 chamber views, for discrimination between HCM and hypertensive LV hypertrophy, as -10.6%; this value was associated with a sensitivity, specificity, and predictive accuracy of 85, 100, and 91.2%, respectively. Similarly, an intraventricular septal/posterior wall thicknesses ratio of 1.3 was associated with a sensitivity of 65%, specificity of 100%, and predictive accuracy of 79.4%. A discriminant function test revealed that a discriminant score (Z) defined by the following equation yielded the highest discriminant probability of 96.1%: Z = -1.7044 + (15.2316 × IVST/PWT) + (1.52687 × strain), where Z > 0 indicates a diagnosis of HCM and Z < 0 indicates a diagnosis of hypertensive LV hypertrophy.

Strain rate imaging has been involved in the differentiation of HCM from cardiac amyloidosis in one study where, however, patients with amyloidosis were in the late stage characterized by low ejection fraction. This suggests that the early differentiation of amyloidosis versus HCM using strain (i.e., before the development of systolic dysfunction) may be still difficult [74].

#### Real time 3-dimensional echocardiography

Real time 3-dimensional echocardiography has been applied to determine LV mass, but there is a paucity of data about its accuracy in HCM. To date, MWT remains the best and the more simple and important measurement that should be reported, because it can predict sudden cardiac death in this population.

#### Coronary flow reserve

Stress echocardiography with dypiridamole has been used to test prognostic role of electrographic signs of inducible ischemia in patients with HCM. In one paper [75], ECG signs of myocardial ischemia elicited by dipyridamole were frequent identify patients at higher risk of cardiac major and minor events, suggesting a important pathogenetic role of inducible myocardial ischemia in determining adverse cardiac events in these patients.

Quantitative evaluation of coronary flow reserve studied by positron emission tomography is a strong predictor of progression to severe symptoms and to ES HCM [76]. An estimate of coronary flow reserve can be obtained in the echo-lab by the simple transthoracic Doppler echocardiographic approach of the mid-distal left anterior descending artery and recently its prognostic role has been tested in a population of patients with HCM. Cortigiani et al [77], found that patients with reduced coronary flow reserve (≤2) have a worse prognosis than patients with normal values (>2). Clinical events recorded during follow-up were death, nonfatal myocardial infarction (defined by typical symptoms, increased cardiac enzyme, and/or electrocardiographic changes), cardioverter- defibrillator implantation, hospitalization for heart failure or unstable angina, syncope, and paroxysmal or chronic atrial fibrillation. Authors found that this coronary flow reserve was a strong and independent predictor of outcome in HCM patients.

#### MRI

MRI is an important imaging technique with an expanding role in the contemporary evaluation of patients with HCM; it provides complete LV reconstruction and a precise definition of the distribution and pattern of hypertrophy [78]. This is particularly useful in patients without a clear LV anatomic characterization by echocardiography. Measurements of MWT by echocardiography and by MRI are strictly related whereas MWT shows weak relationship when related to LV mass [79,80].

In patients highly suspected to have HCM, but with a negative echocardiogram for LV hypertrophy, MRI represents an additive diagnostic chance as proposed by Rickers et al; Authors found, in a population involving almost 50 patients, that MRI was capable of identifying regions of LV hypertrophy (in particular at anterolateral level) not readily recognized by echocardiography, which were solely responsible for diagnosis of the HCM phenotype in an important minority of patients [81]. Moreover, the measure of LV mass and the characterization of abnormal substrate of fibrosis will probably provide implications of these findings in the risk stratification [81]. Nevertheless, it must be underscored that MRI doesn't provide complete LV tissue characterization and, thus, can not be used as a non-invasive biopsy.

### Genetic testing

The diagnosis of HCM is most easily and reliably established by clinical and instrumental examination in the majority of affected adult patients. Thus, in patients with certain clinical diagnosis, genetic testing represents only a diagnostic confirmation. Nevertheless, molecular studies have the potential to enhance diagnostic reliability in HCM and can play an important role in resolving ambiguous diagnoses [82]. Moreover, in selected pedigrees genetic testing, has led to the identification of increasing numbers of children and adults with a preclinical diagnosis of HCM. These individuals have a disease-causing genetic mutation but no clinical or phenotypic manifestations of HCM. At present, there is no available evidence to justify precluding such genotype-positive, phenotype-negative individuals from most employment opportunities or life activities; however, a family history of frequent HCM-related death or the documentation of a particularly malignant genotype may justify efforts at risk stratification and possible restriction from competitive sports. Such a clinical scenario suggest that it is extremely important that family members receive careful counseling both before and after testing [83].

## Conclusion

Two-dimensional echocardiography has been the most used, efficient and accessible technique for establishment of the diagnosis of HCM. Echocardiography can provide important information for the appropriate diagnosis and pathophysiological assessment of HCM patients. However, echocardiography alone can not differentiate different forms of unexplained LV hypertrophy. It must be underscore that its role is highlight only when is used after a complex clinical evaluation including familial and personal anamnesis, clinical examination, electrocardiogram, haemato-chemical tests.

## Competing interests

The authors declare that they have no competing interests.

## Authors' contributions

MAL conceived the review and drafted the manuscript. SN e MG suggested the scheme of the review and revised critically the paper. SB revised critically the manuscript and added figures which resulted in a more readable manuscript. FC and IO revised critically the manuscript and gave important changes before the final submission. EA and PB performed a statistical analysis when necessary and revised critically part of the bibliography giving important criticism on the prognostic role of echo in patients with HCM. SB and ADA helped in the collection of the bibliography suggesting some important papers reported in the review and improved the chapters concerning the differential diagnosis in patients with HCM. ADE and DM contributed and improved the chapters concerning the role of new technologies. SS participated in the design of the review. SM participated in the design of the review and gave the final approval. All authors read and approved the final manuscript.
